# The impact of positioning errors on the dose distribution of hypofractionated radiotherapy for left-sided radical breast cancer

**DOI:** 10.3389/fonc.2026.1805689

**Published:** 2026-04-24

**Authors:** Pan Wang, Guofeng Ma, Ting Zhao, Wei Ding, Wei Kong, Hongqiang Ye, Jun Shang, Wanfu Yang

**Affiliations:** Department of Radiation Oncology, General Hospital of Ningxia Medical University, Yinchuan, Ningxia, China

**Keywords:** dose distribution, hypofractionated radiotherapy, left-sided radical breast cancer, setup errors, thyroid dose

## Abstract

**Purpose:**

To evaluate the impact of setup errors on the dosimetry of targets and organs at risk (OARs) in the mode of Hypofractionated radiotherapy(HFRT) for left-sided radical breast cancer, particularly on the esophagus, thyroid and left anterior descending artery (LAD).

**Methods:**

30 patients were prescribed with 43.5Gy in 15 fractions. Positioning uncertainties were simulated by shifting the isocenter of 3, 5, 7, and 9 mm in six directions. Deviations in dosimetry were analyzed for the supraclavicular and the chest wall clinical target volume (CTV_sc_ and CTV_cw_), the heart, the LAD, the esophagus, and the thyroid under different setup errors.

**Results:**

For D_95%_ (dose covering 95% of the target volume), the CTV_sc_ and CTV_cw_ were most sensitive to errors in the superior (S) and anterior (A) directions, respectively. As the positioning error increased from 3 to 9 mm, the decrease in D_95%_ for CTV_sc_ in the S direction increased from 0.40 Gy to 4.49 Gy, while that for CTV_cw_ in the A direction rose from 0.83 Gy to 11.26 Gy. Among OARs, the dose increase in the esophagus and the thyroid were most pronounced in the right (R) direction, while other OARs such as the heart and LAD were most pronounced in the posterior (P) direction. At a 3 mm error in the most sensitive direction, the D_mean_ (mean dose) of the esophagus, thyroid, LAD, heart, and ipsilateral lung increased by 2.24 Gy, 1.28 Gy, 1.57 Gy, 0.26 Gy, and 0.61 Gy, respectively. When the error increased to 9 mm, these increments further rose to 7.20 Gy, 4.59 Gy, 5.60 Gy, 1.29 Gy, and 2.34 Gy.

**Conclusion:**

The esophagus and thyroid are most sensitive to setup errors in the right direction, while the heart, LAD, and lung are most sensitive to errors in the posterior direction. It is recommended that the setup error tolerance be set at 3 mm for the CTV_cw_ and OARs, whereas for the CTV_sc_ may be relaxed to 5 mm.

## Introduction

Breast cancer is one of the most common malignancies globally among women (1). As a critical component of multidisciplinary breast cancer treatment, radiotherapy plays a pivotal role in reducing local recurrence rates and improving survival outcomes (2). In recent years, with advancements in radiation therapy technology and accumulating clinical evidence (3–5), hypofractionated radiotherapy (HFRT) has gradually emerged as the mainstream modality for breast cancer radiotherapy due to its shorter treatment duration, higher dose per fraction, and enhanced biological effectiveness.

However, the increase in single-fraction dose in HFRT may amplify the impact of potential uncertainties on dose distribution during treatment, among which patient setup errors represent a critical and non-negligible issue in clinical practice (6–9). Setup errors originate from minor deviations in patient positioning, organ motion, and immobilization devices during treatment, which may lead to insufficient target dose coverage or increased radiation exposure to organs at risk (OARs), thereby affecting therapeutic efficacy and potentially inducing radiation-related complications (10–14). While image-guided radiation therapy (IGRT) can partially correct setup errors (15), daily variations in patient positioning remain inevitable even with image guidance. Moreover, daily imaging procedures may increase radiation exposure to OARs, potentially elevating the risk of secondary cancer development (16–18), which is why they are not routinely performed on a daily basis.

Although previous studies have investigated the dosimetric impact of setup errors in conventional fractionated radiotherapy (50 Gy/25 fractions) for breast cancer (19–22), there is currently insufficient systematic analysis of the sensitivity to setup errors and dose deviation characteristics under the HFRT regimen (43.5 Gy/15 fractions). Furthermore, current research predominantly focuses on the dose effects of setup errors on target volumes, heart, and lungs, with critically inadequate attention to esophageal and thyroid exposures.

Based on this, the current study by simulating setup errors, systematically investigate the impact of directional and magnitude variations in setup errors on dose distribution to target volumes and OARs, particularly the esophagus and thyroid, under HFRT. The research results aim to address the insufficient understanding of positioning errors in terms of their impact on dose distribution for the esophagus and thyroid in existing literature. At the same time, provide data support for imaging guidance strategies in fractionated radiotherapy, thereby enhancing treatment efficiency while ensuring the long-term safety of patients.

## Materials and methods

### Patients

A retrospective analysis was performed on 30 patients who underwent radical mastectomy on the left side of our hospital. The inclusion criteria were as follows: ① The primary lesion was located in the left breast and diagnosed as breast cancer by biopsy and pathology; ② Had undergone mastectomy and axillary lymph node dissection without supraclavicular and internal mammary lymph node metastasis; ③ Postoperative adjuvant radiotherapy was performed; ④ Patients could receive neoadjuvant chemotherapy.

The median age of the 30 patients included in the study was 54 years, of which 15 patients received neoadjuvant chemotherapy and 1 patient developed distant metastases. The clinical and tumor characteristics of the 30 patients are shown in **Table 1**.

**Table 1:** Clinical and tumor characteristics of the entire cohort.

pT-stage 0:1:2	9^n^:7^n^:14	
pN-stage 0:1:2	8:17:5	
pM-stage 0:1	28:2	
Parameter	Median	Range
Age(years)	54	33-73
Volume	Mean	standard deviation
CTV_sc_ (cm^3^)	186.98	27.21
CTV_cw_ (cm^3^)	236.75	92.13
Esophgus (cm^3^)	8.14	1.59
Thyroid (cm^3^)	14.17	5.89
Heart (cm^3^)	601.17	81.74
LAD (cm^3^)	7.93	2.58
ipsilateral lung (cm^3^)	1027.50	265.84

“n” represents patients receiving neoadjuvant chemotherapy.

### Patient positioning

The scan device is Siemens Big Bore computed tomography (CT) (SOMATOM Sensation). All patients were immobilized using a dedicated breast board (Klarity) in the supine position, with arms positioned on customized armrests and hands elevated above the head. The support panel angle was adjusted to ensure the chest wall remained parallel to the CT couch during simulation scanning. Then underwent enhanced CT scanning under free breathing condition. The scanning thickness was set to 3mm, and the scanning range covered the mastoid process to the lower margin of the diaphragm.

### Delineation of targets and OARs

**Figure 1:**
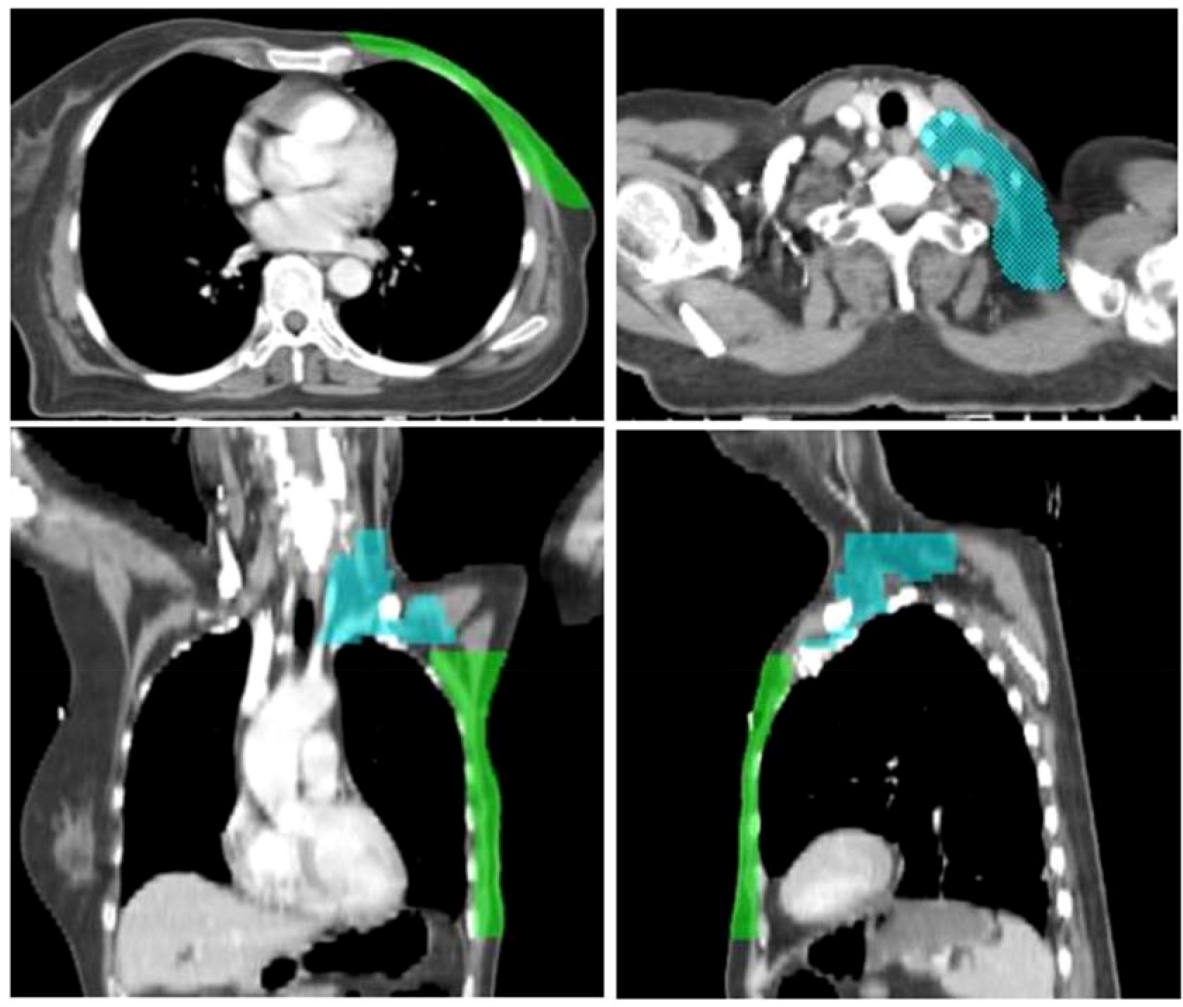
A schematic diagram of CTV_cw_ and CTV_sc_ (green contour for CTV_cw_, sky blue contour for CTV_sc_). CTV_sc_, the clinical target volume of the left supraclavicular region; CTV_cw_, the clinical target volume of the chest wall.

Targets and OARs were delineated according to the protocol (23) by the experienced radiation oncologists. The delineation range of the clinical target volume of the left supraclavicular region (CTV_sc_) is as follows (**Figure 1**): the superior border is at the level of the cricothyroid membrane, the inferior border is at the inferior margin of the left clavicular head, encompassing the left neck levels III and IV as well as the supraclavicular lymphatic drainage area. The delineation range of the clinical target volume of the chest wall (CTV_cw_) is as follows (**Figure 1**): the superior border is at the inferior margin of the left clavicular head, the inferior border is 2cm below the inframammary fold on the healthy side, the medial border is the midline of the body, and the lateral border is the left axillary midline, without exceeding the skin edge and the outer edge of the left lung. The planning target volume of the supraclavicular region (PTV_sc_) and the chest wall (PTV_cw_) were generated by a 5 mm isotropic expansion of the CTV_sc_ and CTV_cw_, respectively, in which PTV_cw_ was retracted to the edge of the skin and the outer edge of the left lung. It is worth noting that the esophagus was delineated from the lower border level of the cricoid cartilage to the lower margin of the aortic arch. This is because previous studies (24, 25) have confirmed that using dosimetric parameters of the upper esophagus is more suitable for assessing the radiation dose to the esophagus. The delineation of targets and OARs was reviewed and confirmed by an experienced superior physician.

### Radiotherapy planning

In this study, the Pinnacle 16.2 version (Philips Medical Systems) treatment planning system was used to design radiotherapy plans. The plan was calculated using the adaptive convolve algorithm and a grid calculation size of 3 mm. The radiotherapy plan employs multibeam inverse planned intensity modulated RT (IMRT) with a 6 MV beam and the beam angle may vary slightly depending on the size of the patient body and target volume. Specifically, there were two tangential chest wall fields (300^0^-310^0^ and 115^0^-125^0^), two supraclavicular fields (330^0^-340^0^ and 20^0^-30^0^), and one composite field (100°- 110°) designed to concurrently irradiate the partial chest wall and supraclavicular lymph node region. All enrolled patients received a prescription dose of 43.5Gy in 15 fractions. The dose constraints are as follows (23): at least 95% of the PTV_sc_ and PTV_cw_ receiving 43.5 Gy and 95% of the prescription dose covering at least 99% of the PTV_sc_ and PTV_cw_; the maximum dose(D_max_) to the PTV_sc_ and PTV_cw_ should not exceeding 110% of the prescription dose; D_max_ of esophagus should not exceeding 48Gy; the mean dose (D_mean_) of the thyroid should not exceeding 21 Gy; less than 30% of the ipsilateral lung to 20 Gy and D_mean_ not exceeding 15 Gy; D_mean_ of the heart should not exceeding 6 Gy; less than 20% of the left anterior descending artery (LAD) to 30 Gy. Since the CTV_cw_ encompasses the skin, a 0.5cm thick tissue-equivalent bolus was applied to account for the dose build-up effect, thereby optimizing skin dose homogeneity. All radiotherapy plans should be reviewed by superior physicists and physicians.

### Simulation of set-up uncertainty

We simulated the set-up uncertainty by shifting the isocenter from its reference position in the left(L), right(R), superior(S), inferior(I), anterior(A) and posterior(P) direction. Based on the actual setup error data (**Table 2**), the simulated setup errors were designed to be 3, 5, 7, and 9 mm. The dose distribution of perturbed plan was recalculated without changing the monitor units. A total of 24 plans were generated for each patient based on the original plans, and a total of720 plans were generated for all patients.

**Table 2:** The actual setup errors of patients (cm).

Parameter	L/R	S/I	A/P
Max	0.04	0.02	0.02
Min	-1.06	-1.25	-1.11
M	-0.23	-0.20	0.29
SD	0.41	0.42	0.32
∑	0.24	0.23	0.21
σ	0.43	0.44	0.34

Max, the maximum; Min, the minimum; M, the mean; SD, the standard deviation; ∑, the systematic error; σ, the random error; L, the left; R, the right; S, the superior; I, the inferior; A, the anterior; P, the posterior.

### Statistics

Compare the dose deviations in organs at risk (OARs) and target volumes between the original treatment plan and the simulated plan, and the data were presented as mean ± SD or median (25th percentile, 75th percentile). The Shapiro-Wilk test was used to verify the normality of the data. If the data followed a normal distribution, the paired sample t-test was employed. If the data did not follow a normal distribution, the Wilcoxon rank sum test was adopted. P < 0.05 indicated that the difference was statistically significant.

## Result

### The original plan characteristics

The statistical data of original plan are shown in **Table 3**. D_X%_ represents the dose received by X% of the volume; RV_XGy_ represents the relative volume receiving X Gy; D_mean_ represents the mean dose.

**Table 3:** The dose parameters of targets and OARs in the original plan.

Structure	Parameter	Median	(25th percentile, 75th percentile)
CTV_sc_	D_95%_(Gy)	43.38	43.25, 43.25
	D_2%_(Gy)	46.31	46.17, 46.41
CTV_cw_	D_95%_(Gy)	43.32	43.20, 43.44
	D_2%_(Gy)	46.39	46.14, 46.59
Esophagus	RV_25_(%)	37.22	29.92, 55.09
	D_mean_(Gy)	19.51	17.00, 24.69
Thyroid	RV_30_(%)	40.12	35.09, 47.08
	RV_45_(%)	12.68	10.09, 19.00
	D_mean_(Gy)	25.41	23.48, 26, 58
Heart	D_mean_(Gy)	1.80	1.38, 2.46
LAD	RV_30_(%)	6.36	0.03, 21.55
	D_mean_(Gy)	8.10	4.09, 13.55
ipsilateral lung	RV_20_(%)	17.36	15.64, 18.64
	D_mean_(Gy)	8.50	8.02, 9.14

Data are presented as median (25th percentile, 75th percentile). Since the data were not normally distributed, the median and interquartile range (IQR) are used to describe the central tendency and statistical dispersion.

### Variation of targets

The detailed data of target dose deviation △X are shown in **Table 4**. It can be observed that the impact of setup errors on the D_95%_ is greater than on D_2%_.

**Table 4:** Dose deviations △X (Gy)of D_95%_ and D_2%_ between perturbed and original plans.

Errors	CTV_sc_		CTV_cw_	
	D_95%_	D_2%_	D_95%_	D_2%_
3mm				
L	-0.21 (-0.32, -0.13)	0.02 (-0.03, 0.07)	-0.41 (-0.48, -0.27)	0.15 (0.08, 0.20)
R	-0.06 (-0.17, 0.01)	-0.08 (-0.14-0.01)	-0.09 (-0.13, 0.07)	-0.18 (-0.23, -0.11)
A	-0.21 (-0.31, -0.12)	0.05 (-0.08, 0.16)	**-0.83 (-0.98, -0.63)**	0.26 (0.17, 0.31)
P	-0.05 (-0.11, 0.04)	-0.10 (-0.19, -0.06)	-0.16 (-0.44, -0.06)	-0.31 (-0.37, -0.23)
S	**-0.40 (-0.54, -0.21)**	-0.03 (-0.04, 0.02)	-0.20 (-0.27, -0.15)	0.01 ()-0.03, 0.04
I	-0.29 (-0.34, -0.17)	0.00 (-0.06, 0.04)	-0.01 (-0.05, 0.05)	-0.03 (-0.12, 0.01)
5mm				
L	-0.83 (-1.07, -0.52)	0.12 (0.03, 0.17)	-1.11 (-1.29, -0.85)	0.36 (0.28, 0.44)
R	-0.32 (-0.47, -0.21)	-0.19 (-0.23-0.125)	-0.56 (-0.82, -0.21)	-0.34 (-0.45, -0.28)
A	-0.55 (-0.73, -0.39)	0.17 (-0.10, 0.32)	**-2.78 (-3.01, -2.41)**	0.52 (0.42, 0.62)
P	-0.23 (-0.35, -0.15)	-0.20 (-0.34, -0.09)	-1.33 (-2.28, -0.86)	-0.58 (-0.70, -0.43)
S	**-1.10 (-1.51, -0.82) **	-0.06 (-0.12, -0.02)	-0.58 (-0.78, -0.43)	0.06 (-0.01, 0.12)
I	-0.82 (-1.28, -0.51)	0.05 (-0.07, 0.09)	-0.03 (-0.17, 0.02)	-0.03 (-0.13, 0.06)
7mm				
L	-1.64 (-2.56, -1.28)	0.23 (0.12, 0.27)	-2.21 (-2.93, -1.80)	0.54 (0.44, 0.66)
R	-0.78 (-1.08, -0.59)	-0.28 (-0.35-0.18)	-1.33 (-1.88, -0.71)	-0.55 (-0.65, -0.43)
A	-1.14 (-1.32, -0.81)	0.25 (-0.09, 0.49)	**-6.12 (-6.53, -5.58)**	0.78 (0.64, 0.87)
P	-0.59 (-0.69, -0.44)	-0.28 (-0.52, -0.14)	-3.53 (-5.52, -2.63)	-0.88 (-1.04, -0.69)
S	**-2.33 (-3.22, -1.71) **	-0.07 (-0.15, -0.01)	-1.33 (-1.53, -1.12)	0.07 (0.01, 0.17)
I	-1.91 (-3.19, -1.40)	0.04 (-0.09, 0.13)	-0.24 (-0.42, -0.16)	-0.01 (-0.14, 0.10)
9mm				
L	-3.06 (-4.38, -2.47)	0.30 (0.21, 0.35)	-3.91 (-5.30, -3.06)	0.73 (0.58, 0.86)
R	-1.49 (-2.25, -1.06)	-0.35 (-0.47-0.24)	-2.68 (-3.55, -1.44)	-0.76 (-0.88, -0.65)
A	-1.93 (-2.12, -1.34)	0.34 (-0.08, 0.63)	**-11.26 (-12.14, -10.11)**	0.99 (0.66, 1.16)
P	-1.09 (-1.32, -0.90)	-0.38 (-0.72, -0.20)	-7.16 (-10.34, -6.69)	-1.14 (-1.48, -0.88)
S	**-4.49 (-5.97, -3.32) **	-0.09 (-0.22, -0.01)	-2.37 (-2.71, -2.14)	0.10 (0.04, 0.18)
I	-3.93 (-5.47, -3.43)	0.05 (-0.09, 0.14)	-0.69 (-0.92, -0.41)	-0.02 (-0.20, 0.15)

Bold text indicates the direction with the greatest change in dosage. △X = perturbed plans – original plans. Data are presented as median (25th percentile, 75th percentile). Since the data were not normally distributed, the median and interquartile range (IQR) are used to describe the central tendency and statistical dispersion.

In terms of D_95%_, the dose sensitivity to setup errors is higher for CTV_cw_ than for CTV_sc_. Specifically, when setup errors were ≤5 mm, the median dose reduction for CTV_sc_ did not exceed 2 Gy in any direction, while for CTV_cw_, it reached 2.78 Gy in the A direction. When errors increased to 7 mm, the median dose reduction for CTV_sc_ was 2.33 Gy in the S direction, whereas for CTV_cw_, it reached 6.12 Gy, 3.53Gy, and 2.21 Gy in the A, P, and L directions, respectively. When errors reached 9 mm, the median dose reduction for CTV_sc_ was 4.49 Gy, 3.93 Gy, and 3.06 Gy in the S, I, and L directions, respectively. In contrast, for CTV_cw_, the dose change exceeded 2 Gy in all directions except I direction, with the median dose reduction notably reaching 11.26 Gy in the A direction.

**Figure 2:**
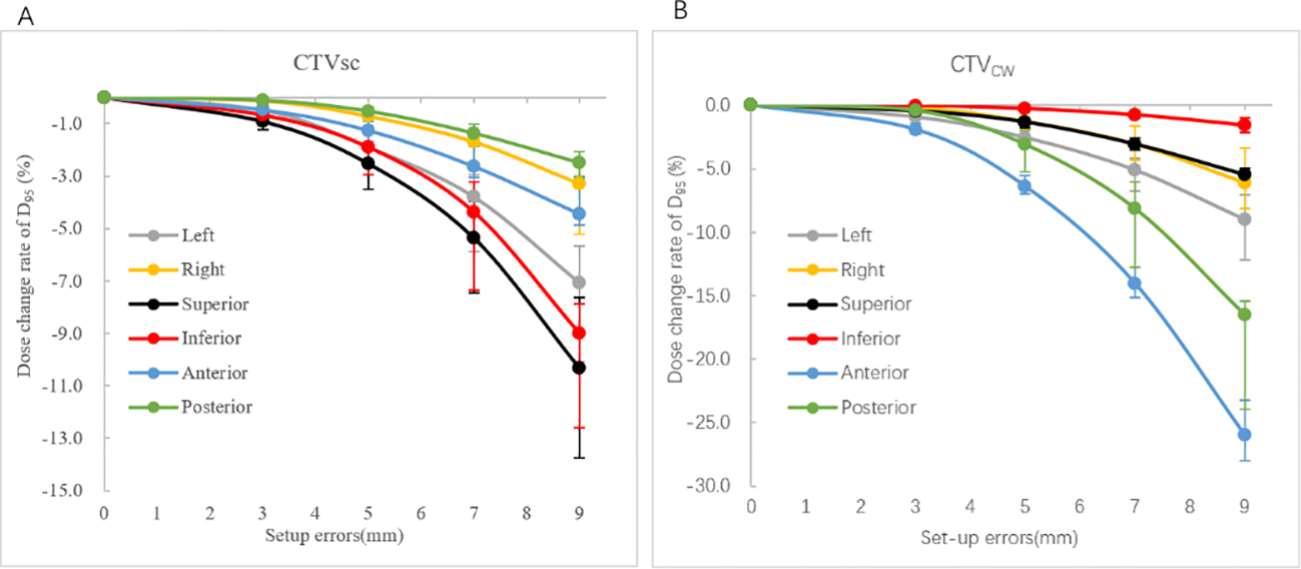
Dose change rate of targets under setup errors in different directions and magnitudes. Error bars represent interquartile range (IQR). **(A)** represents the dose change rate of D95% for CTVSC. **(B)** represents the dose change rate of D95% for CTVCW.

**Figure 3:**
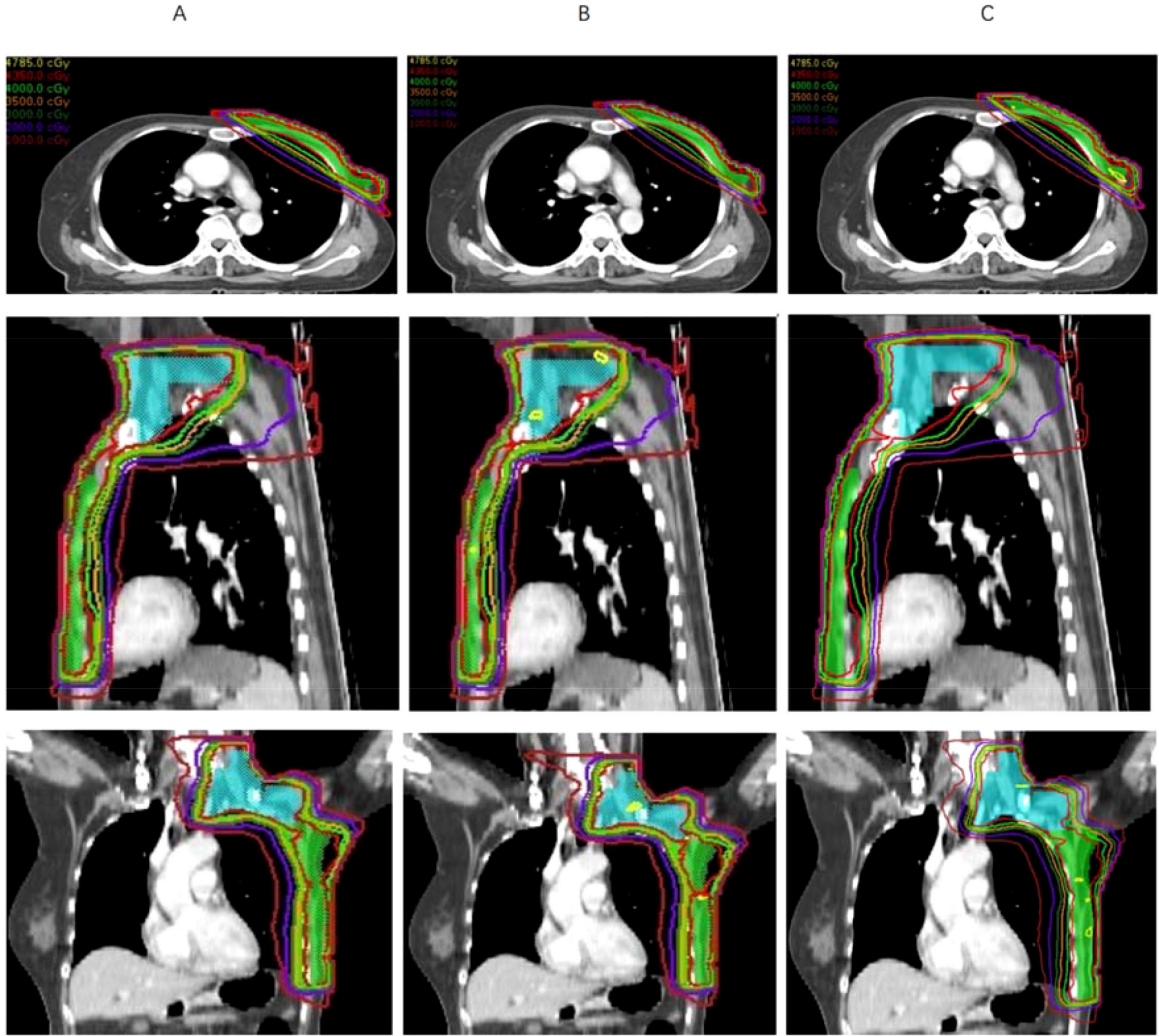
Dose distribution of the original plan and perturbed plan for a representative case. **(A)** Dose distribution of the original plan. **(B)** Dose distribution of the perturbed plan in the superior direction with 5mm setup errors. **(C)** Dose distribution of the perturbed plan in the anterior direction with 5mm setup errors.

**Figure 2** presents the dose variation rates of the target volumes under setup errors of different directions and magnitudes. The results showed that the D_95%_ of the CTV_sc_ and CTV_cw_ exhibited different sensitivities to setup errors in different directions. Specifically, the D_95%_ for CTV_sc_ was most sensitive to setup errors in the S direction and least sensitive to those in the P direction. In contrast, the D_95%_ for CTV_cw_ was most sensitive to setup errors in the A direction and least sensitive to those in the I direction. To visually demonstrate the dose variations in the S and A directions for CTV_sc_ and CTV_cw_, **Figure 3** presents a comparison of the original plan and the simulated dose distribution for one patient case.

### Variations of OARs

**Supplementary Table 1** details the dose deviations for various OARs across six directions. The results indicate that setup errors in the R, P, and I directions led to increased radiation doses to the esophagus, heart, left anterior descending artery (LAD), and ipsilateral lung. Conversely, elevated radiation doses to the thyroid were observed only when setup errors occurred in the R, A, and S directions.

**Figure 4:**
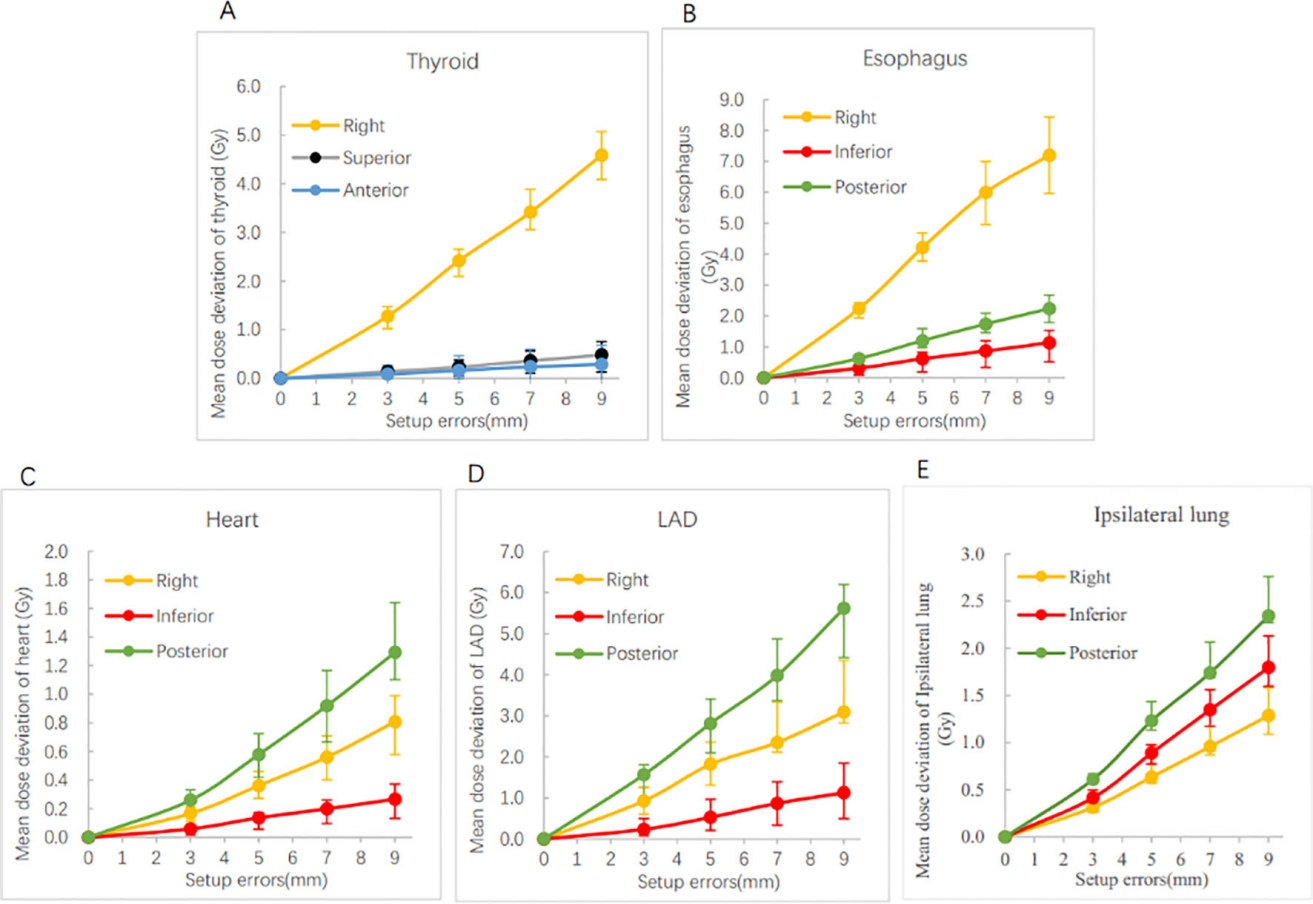
Dose variation in mean dose of organs at risk in the direction of dose increase. LAD: left anterior descending artery. Error bars represent interquartile range (IQR). **(A)** represents the dose variation of thyroid in the direction of right, superior , and anterior. **(B)** represents the dose variation of esophagus in the right, inferior, and posterior. **(C)** represents the dose variation of heart in the right, inferior, and posterior. **(D)** represents the dose variation of LAD in the right, inferior, and posterior. **(E)** represents the dose variation of ipsilateral lung in the right, inferior, and posterior.

**Figure 4** shows the variations in doses to OARs resulting from setup errors along the dose−increasing direction. It can be clearly seen that setup errors in the right (R) direction had the most pronounced impact on the doses to the esophagus and thyroid, while errors in the posterior (P) direction most significantly affected the doses to the heart, LAD, and ipsilateral lung. Specifically, when the setup error was 3 mm, the mean doses to the esophagus and thyroid increased by 2.24Gy and 1.28 Gy, respectively, in the R direction; in the P direction, only the mean dose to the LAD increased by more than 1 Gy. When the error reached 9 mm, the mean dose increases for the esophagus and thyroid in the R direction rose to 7.20 Gy and 4.59 Gy, respectively. Concurrently, the mean dose increases for the LAD, ipsilateral lung, and heart in the P direction all exceeded 1 Gy.

To evaluate the impact of combined setup errors on OARs, we adopted a worst-case scenario based on the sensitivity of OARs to setup errors in different directions. Specifically, we assessed the effect of combined setup errors only in the most dose-sensitive directions for each organ. **Supplementary Table 2** presents the impact of setup errors of various magnitudes for the combination of the R, P, and I directions on the esophagus, LAD, heart, and lung, but for the thyroid, the combination of directions is R, A, and S. As shown in the table, combined errors significantly increase the dose impact on organs at risk compared with single−direction errors.

**Figure 5:**
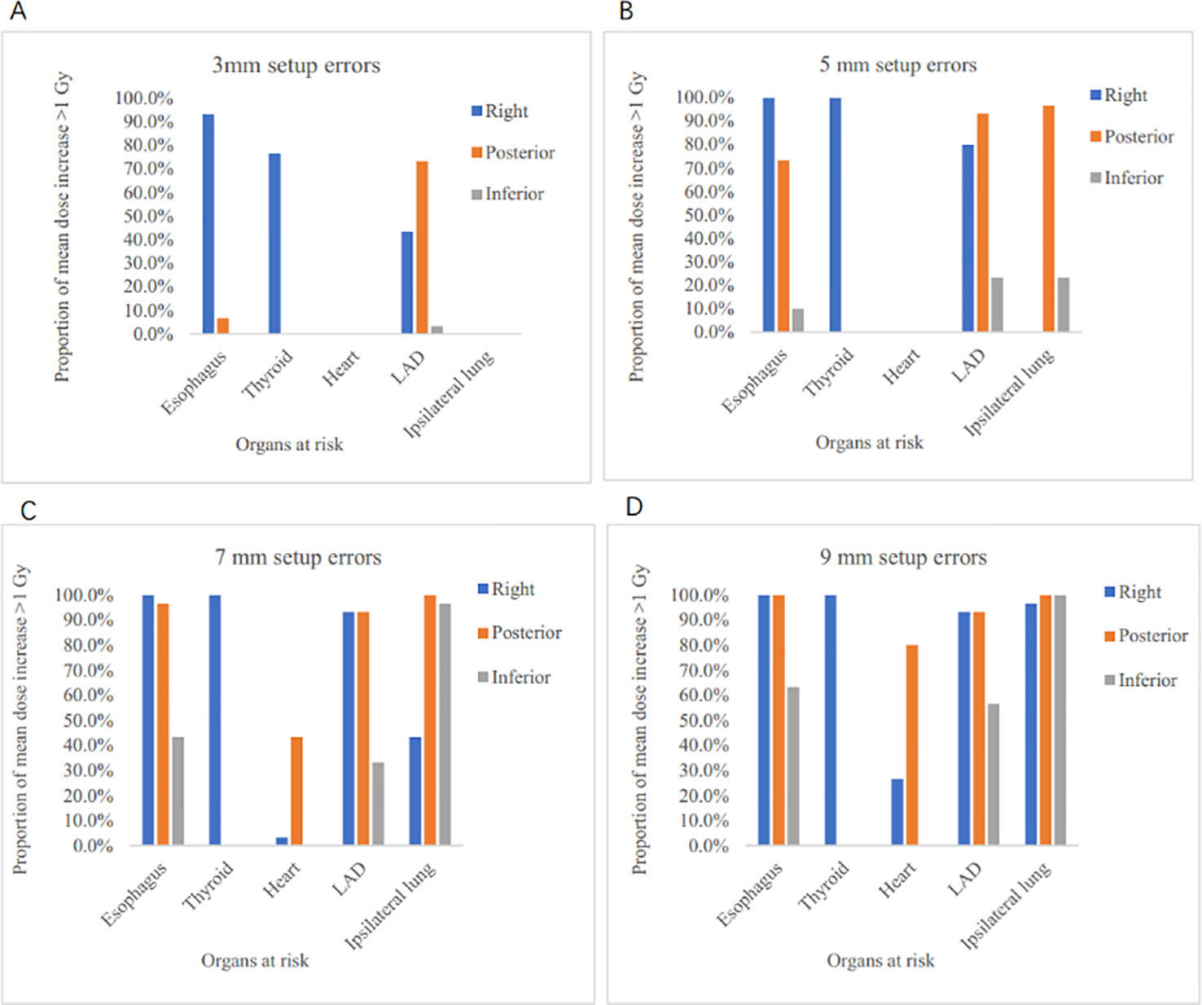
Proportion of organs at risk with mean dose increase exceeding 1 Gy caused by setup errors of varying magnitudes, stratified by the R, P, and I directions, respectively. **(A)** represents the proportion of organs at risk with a mean dose increase >1 Gy in the R, P, and I directions under a 3 mm setup error. **(B)** represents the proportion of organs at risk with a mean dose increase >1 Gy in the R, P, and I directions under a 5 mm setup error. **(C)** represents the proportion of organs at risk with a mean dose increase >1 Gy in the R, P, and I directions under a 7 mm setup error. **(D)** represents the proportion of organs at risk with a mean dose increase >1 Gy in the R, P, and I directions under a 9 mm setup error.

**Figure 6:**
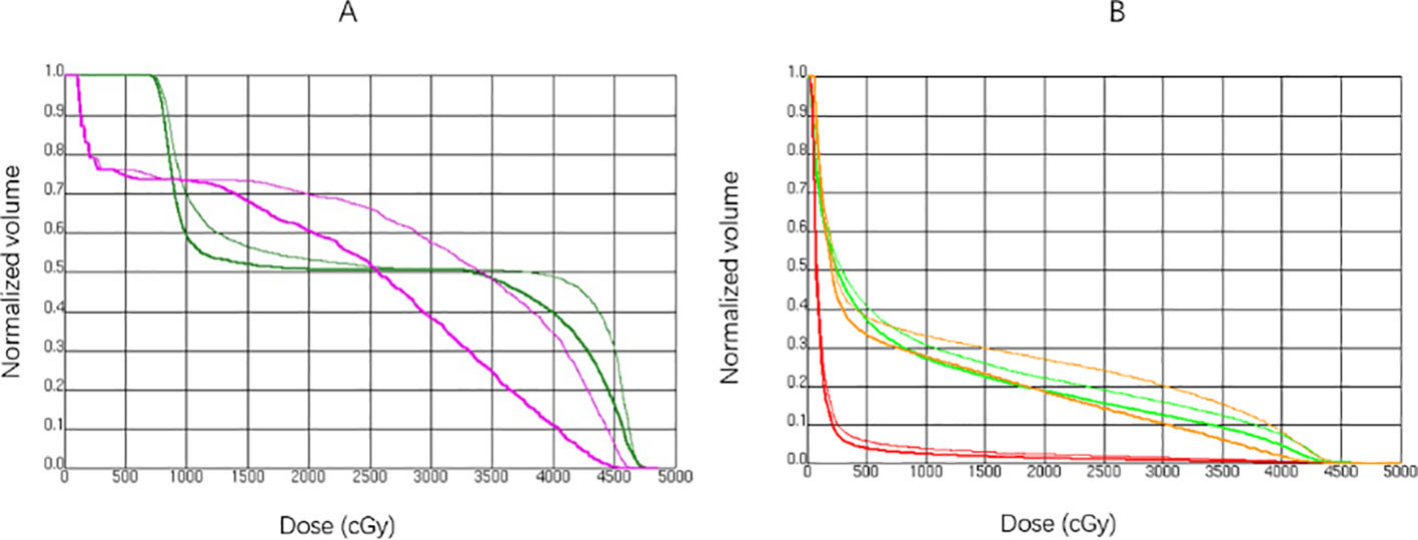
The dose volume histogram(DVH) of OARs in a representative case. The thick solid line represents the DVH for OARs in the original plan, while the thin solid line represents the DVH after introducing a 5 mm setup error. **(A)** The DVH for the esophagus (magenta) and thyroid (forest green) in the R direction. **(B)** The DVH for the heart (red), left anterior descending artery (LAD, orange), and ipsilateral lung (green) in the S direction.

**Figure 5** shows, for the R, P, and I directions, the proportion of OARs for which the mean dose increment exceeds 1 Gy due to setup errors of different magnitudes. As shown in the figure, at setup errors ≤3 mm, no mean dose increase >1 Gy occurred for the heart or ipsilateral lung. In the R direction, the incidence of >1 Gy increase was 93.3% for the esophagus, 76.7% for the thyroid, and 43.3% for the LAD. As errors increased, dose increments became more prominent, especially for the ipsilateral lung. At 9 mm, the incidence reached 100% for the ipsilateral lung in the P and I directions. **Figure 6** displays the dose distribution maps for OARs in a representative patient, comparing the original plan with the plan simulated under setup errors.

## Discussion

This study systematically analyzed the impact of setup errors in six cardinal directions on radiotherapy dose distributions, revealing distinct dosimetric variation patterns in target volumes and OARs (particularly the esophagus and thyroid). The results demonstrate that setup errors cause significant dose reduction in D_95%_, with substructural differences in dose stability observed between clinical target volumes: the CTV_sc_ exhibited superior dose stability compared to the CTV_cw_. Further analysis revealed a dual nature of dose changes in OARs: their magnitude correlated with error magnitude while showing pronounced directional dependence. Only errors in specific directions led to increased doses in OARs, and directional sensitivity to spatial errors varied among different OARs.

For the D_95%_, within a 5 mm setup error range, CTV_sc_ exhibited excellent dose maintenance capability, with dose variations caused by setup errors in any direction not exceeding 3%. In contrast, CTV_cw_ decreased by 6.40% and 3.04% in the A and P directions, respectively. This marked difference in dose variation is attributed to the different methods of PTV generation: PTV_sc_ was generated by uniformly expanding CTV_sc_ by 5 mm, which effectively maintains target dose coverage within a certain setup error range. In contrast, PTV_cw_ was generated by expanding CTV_cw_ by 5 mm and then trimming to the skin surface, leaving insufficient expansion margin in the A direction to ensure dose stability. This finding is similar to that of Zhao Y (26), where a 5 mm setup error significantly affected target dose due to the use of a 3 mm expansion margin. This phenomenon highlights the critical role of appropriately setting expansion margins to balance target coverage (27, 28); an appropriate margin not only reduces the risk of target underdose but also minimizes the likelihood of unnecessary irradiation of surrounding normal tissues.

For patients with radical breast cancer, the target volume is typically large, encompassing both the chest wall and supraclavicular region, which increases the complexity of setup and makes setup errors more likely to occur. Based on the differential sensitivity of CTV_sc_ and CTV_cw_ to setup errors of varying magnitudes, we recommend a setup error tolerance of 3 mm for CTV_cw_ and 5 mm for CTV_sc_. This difference in tolerances suggests that, in routine clinical practice, When the registration deviations of CTV_sc_ and CTV_cw_ differ during image guidance, priority should be given to online correction using CTV_cw_.

Relevant studies (7, 29) indicate that patients undergoing modified radical mastectomy exhibit the most significant chest wall movement in the A and P directions under free-breathing conditions, with maximum amplitudes reaching 4.2-5.4mm. Combined with the findings of this study, when setup errors in the A and P directions reach 5mm, the CTV_cw_ D_95%_ dose variation exceeds 3%. Previous research (30, 31) has shown that dose variations of 3-5% may affect tumor control probability (TCP) and normal tissue complication probability (NTCP), thus requiring clinical attention to target displacement caused by respiratory motion. A multicenter study (32) reported that SGRT(surface-guided radiation therapy)−guided DIBH(deep inspiration breath hold) achieves systematic errors of <4 mm in translational directions, which, based on our findings, can limit target dose variations to within 5%. Moreover, SGRT has been shown to reduce the frequency of cone−beam CT (CBCT) verification (33),thereby decreasing cumulative radiation exposure and improving workflow efficiency. For institutions where this technology is not yet available, maintaining CBCT verification frequency remains necessary.

This study revealed that the directions significantly affecting D_95%_ for CTV_cw_ and CTV_sc_ were A and S directions, respectively. This sensitivity difference is closely related to target shape and anatomical location. As shown in **Figure 1**, CTV_sc_ exhibits significant morphological variations in the craniocaudal direction, resulting in greater fluctuations in dose conformality within this axis. Consequently, D_95%_ demonstrates heightened sensitivity to setup errors in this direction. Conversely, CTV_cw_ is located in the thoracic region, adjacent to critical organs such as the heart, ipsilateral lung, and LAD, forming distinct high-dose gradient regions. When setup errors occur in the A and P directions, the radiation fields deviate maximally from the target, resulting in significant reductions in target coverage. Notably, research by Xiongfei Liao et al (20) using Volumetric Modulated Arc Therapy (VMAT) identified setup errors in the A and L directions as having the greatest impact on D_95%_, differing from our findings (based on fixed-field intensity-modulated radiation therapy, IMRT). This discrepancy suggests that radiotherapy techniques may influence target sensitivity to setup errors, necessitating further comparative studies to clarify the underlying mechanisms. Based on the differences in directional sensitivity to setup errors between CTV_cw_ and CTV_sc_, we recommend evaluating them separately during target delineation, treatment planning, and IGRT decision-making to better balance target coverage and organs at risk sparing.

Radiation-induced esophagitis, an often-overlooked complication in breast cancer radiotherapy, develops in close association with aberrant esophageal dose exposure caused by setup errors. This study’s three-dimensional error analysis revealed that setup deviations in the R, P, and I directions all increase esophageal radiation dose, with R-direction errors exerting the most significant impact. Anatomic analysis indicates the esophagus lies left of the midline, where its upper segment closely neighbors the CTVsc. Portions of esophageal tissue may even lie within the target volume. When R-direction setup errors occur, additional esophageal tissue enters the radiation field boundary, substantially increasing its dose exposure. Therefore, clinical practice should prioritize monitoring R-direction setup errors to mitigate esophageal dose risks.

The study by Journy et al (34) demonstrated that each 1 Gy increase in esophageal radiation dose was associated with a 7.1% elevated risk of esophageal cancer. According to the findings of the present study, when setup errors in the R direction reached 3 mm and 5 mm, the D_mean_ of esophagus increased by 2.24Gy and 4.21Gy, respectively. Although this study could not precisely quantify the esophageal cancer risk using Journy et al’s model due to incomplete contouring of the entire esophagus, it is noteworthy that increasing R-direction setup errors led to a continuous escalation of esophageal dose, suggesting a concurrent rise in associated risks. Dan-Qiong W (25) reported that RV_25_ was significantly correlated with grade 2 radiation-induced esophagitis, with a marked increase in incidence when RV_25_ exceeded 20%. In our study, the original treatment plan showed an RV_25_ as high as 37.22%, indicating a substantially elevated risk of grade 2 esophagitis. Furthermore, increases in setup errors along the R, P, and I directions resulted in a significant rise in esophageal RV_25_, further elevating the likelihood of grade 2 esophagitis. Therefore, during radiotherapy plan optimization, it is essential to incorporate stricter dose constraints, specifically targeting RV_25_ < 20%, to mitigate the incidence of grade 2 radiation-induced esophagitis.

The D_mean_ of heart is widely recognized as a critical parameter for predicting cardiac toxicity in breast cancer patients. Darby et al (10) demonstrated a linear relationship between increasing D_mean_ and the risk of major coronary events, with a 7.4% relative risk increase per 1 Gy rise in mean dose. According to the findings of this study, when setup errors ≤ 9 mm, the change in mean heart dose (ΔD_mean_) stays below 1 Gy, suggesting limited implications for cardiac toxicity. However, further analysis revealed that the LAD, a key coronary structure, exhibits higher sensitivity to setup errors compared to the heart. Specifically, at a 3 mm setup error, LAD mean dose increased by0.93, 1.57, and 0.24 Gy in the R, P and I directions, respectively.

In fact, existing studies (35–37) have clearly indicated that the mean heart dose should not be the only indicator for assessing cardiotoxicity, and the radiation exposure to the LAD should also be incorporated into the evaluation system. The expert group of DEGRO (37) has proposed specific criteria: mean heart dose < 2.5 Gy, mean LAD dose < 10 Gy, and RV_30_ < 2%. According to the results of this study, when the setup error is 5 mm, the mean dose to the LAD in the P direction reaches 10.91Gy, exceeding the threshold recommended by the DEGRO expert group, whereas the mean heart dose in the R, P, and I directions all remain within the suggested limits. This finding suggests that if only the whole heart is considered in the evaluation, the impact of setup errors on cardiotoxicity is likely to be underestimated. Therefore, during breast cancer radiotherapy, it is essential not only to closely monitor the influence of setup errors on the overall heart dose but also to pay particular attention to their effect on the radiation dose received by the LAD, so as to minimize cardiotoxic damage caused by setup inaccuracies.

This study found that under the influence of setup errors, thyroid radiation exposure exhibits significant directional dependence. When setup errors occur in the R, A, and S directions, the radiation dose to the thyroid increases specifically, with the right directional error having the most pronounced impact on the dose. Research (38) indicates that during breast radiotherapy, particularly when the supraclavicular lymph node region is included, excessive irradiation of the thyroid increases the risk of radiation-induced hypothyroidism (RHT). The prospective cohort study by Xu-Ran Zhao et al (39) confirmed that the mean thyroid dose is an independent risk factor for RHT and recommended controlling D_mean_ below 21 Gy in hypofractionated radiotherapy. Similarly, Haciislamoglu et al (40) suggested a thyroid dose constraint of RV_30_ < 50% and a mean dose < 21 Gy. The mean thyroid dose observed in this study was 21.80 Gy, which is slightly above the aforementioned threshold. However, Dahbi (41) reported an incidence of hypothyroidism of only 4% under hypofractionated radiotherapy.Therefore, we consider this dose level clinically acceptable.

According to the findings of this study, in the original treatment plan, the dose of thyroid and esophagus slightly exceeding the recommended constraints mentioned above. The primary reason for this is that during the planning phase, target coverage was prioritized as the main optimization objective, and part of the thyroid gland and esophagus fell within the target volume. When using IMRT techniques, there are inherent technical limitations in sparing the thyroid and esophagus, resulting in slight dose excess. Several studies (42, 43) have shown that VMAT offers dosimetric advantages over IMRT in thyroid protection. One study (43) comparing hypofractionated breast radiotherapy reported that VMAT achieved significantly lower thyroid RV_30_ and mean dose than IMRT. Regarding esophageal protection, another study (44) revealed different characteristics: IMRT was superior to VMAT in D_max_ of esophagus, but inferior in D_mean_ of esophagus. These findings suggest that the technical limitations of IMRT do not simply imply inferiority to VMAT.

This study is the first to systematically reveal the unique sensitivity of the esophagus and thyroid to positioning errors in hypofractionated radiotherapy for breast cancer. However, our findings are based on IMRT. Previous studies have compared the dosimetric robustness of IMRT and VMAT in breast cancer patients. Zhang Z (45) evaluated setup error effects in 32 postmastectomy patients and found that after accounting for positioning uncertainties, VMAT achieved significantly higher CTV coverage (95.5%) than IMRT (91.3%), indicating better robustness of VMAT against setup errors. Similarly, Yin C (46) quantified plan robustness using DVH bandwidth (DVHBW) in 50 breast cancer patients and demonstrated that VMAT was less sensitive to setup errors. These results suggest that the impact of setup errors varies markedly between techniques. Therefore, our conclusions derived from IMRT cannot be directly generalized to VMAT.

## Conclusion

Setup errors have direction-specific dosimetric effects on targets and OARs. Specifically, CTV_sc_ and CTV_cw_ are most sensitive to errors in the superior and anterior directions, respectively; the esophagus and thyroid to the right direction; and the heart, LAD, and lung to the posterior direction. Based on the findings of this study, a setup error tolerance of 3 mm is recommended for CTV_cw_, while 5 mm is acceptable for CTV_sc_. In clinical practice, differentiated setup error management strategies should be adopted based on the location of the target volume and its directional sensitivity.

## Data Availability

The original contributions presented in the study are included in the article/**Supplementary Material**. Further inquiries can be directed to the corresponding author.
