# Supplementary material for: The impact of positioning errors on the dose distribution of hypofractionated radiotherapy for left-sided radical breast cancer
Source: Front Oncol. 2026 Apr 24;16:1805689. doi: 10.3389/fonc.2026.1805689 (PMC13152794; doi:10.3389/fonc.2026.1805689)
Supplement: Supplementary file 1 [file Table1.docx]

**Supplementary Table1** Dose deviations △X of OARs between perturbed and original plans in the six directions.

| **OARs** | **Parameter** | **3mm** | **5mm** | **7mm** | **9mm** |
| --- | --- | --- | --- | --- | --- |
| **Left** | | | | | |
| Esophagus | △RV_25_(%) | -10.88(-12.26, -9.09) | -20.71(-24.59, -17.19) | -28.14(-35.68, -26.20) | -35.59(-43.93, -29.49) |
|  | △D_m_(Gy) | -2.39(-2.70, -2.11) | -4.67(-5.21, -4.07) | -6.76(-7.49, -5.84) | -8.88(-9.72, -7.24) |
| Thyroid | △RV_30_(%) | -3.58 (-5.49, -2.17) | -8.82 (-11.68, -6.22) | -15.23(-17.65, -12.27) | -21.90(-24.34, -19.16) |
|  | △RV_45_(%) | -5.58(-6.31, -4.03) | -9.34(-11.03, -7.61) | -11.42(-14.14, -9.49) | -11.91(-17.22, -9.91) |
|  | △D_m_(Gy) | -1.32(-1.54, -1.03) | -2.62(-3.11, -2.18) | -4.17(-4.66, -3.36) | -5.44(-6.03, -4.70) |
| Heart | △D_m_(Gy) | -0.21(-0.26, -0.12) | -0.37(-0.46, -0.25) | -0.56(-0.68, -0.37) | -0.77(-0.85, -0.51) |
| Ipsilateral lung | △RV_20_(%) | -1.02(-1.52, -0.72) | -2.05(-2.67, -1.45) | -2.92(-3.40, -2.18) | -3.56(-4.28, -3.04) |
|  | △D_m_(Gy) | -0.32(-0.38, -0.25) | -0.63(-0.73, -0.55) | -0.93(-1.03, -0.83) | -1.24(-1.42, -1.13) |
| LAD | △RV_30_(%) | -2.20(-3.36, -0.03) | -4.24(-6.41, -0.03) | -6.28(-9.33, -0.03) | -7.09(11.78, -0.03) |
|  | △D_m_(Gy) | -1.01(-1.11, -0.57) | -1.73(-2.22, -1.06) | -2.63(-3.21, -1.64) | -3.54(-4.40, -2.28) |
| **Right** | | | | | |
| Esophagus | △RV_25_(%) | 8.17(6.44, 10.49) | 15.76(9.91, 20.1) | 17.76(11.92, 27.56) | 18.93 (13.53, 31.83) |
|  | △D_m_(Gy) | 2.24(1.94, 2.44) | 4.21(3.78, 4.69) | 6.00(4.96, 7.00) | 7.20(5.96, 8.43) |
| Thyroid | △RV_30_(%) | 1.66(0.91, 2.22) | 2.82(2.06, 3.46) | 3.97(2.44, 4.98) | 4.325(2.96, 6.35) |
|  | △RV_45_(%) | 4.80(2.66, 5.78) | 9.38(7.40, 11.26) | 13.63(11.94, 15.83) | 16.48(12.91, 18.86) |
|  | △D_m_(Gy) | 1.28(1.02, 1.47) | 2.42(2.10, 2.65) | 3.41(3.06, 3.89) | 4.59(4.09, 5.07) |
| Heart | △D_m_(Gy) | 0.17(0.11, 0.21) | 0.36(0.27, 0.46) | 0.56(0.40, 0.71) | 0.81(0.58, 0.99) |
| Ipsilateral lung | △RV_20_(%) | 0.71(0.38, 0.87) | 1.46(1.03, 2.06) | 2.29(1.96, 2.85) | 3.16(2.68, 3.77) |
|  | △D_m_(Gy) | 0.31(0.26, 0.42) | 0.63(0.57, 0.86) | 0.96(0.87, 1.18) | 1.29(1.09, 1.58) |
| LAD | △RV_30_(%) | 2.16(0.54, 3.48) | 4.08(1.56, 7.25) | 6.30(3.49, 11.00) | 7.93(4.81, 13.69) |
|  | △D_m_(Gy) | 0.93(0.60, 1.26) | 1.82(1.31, 2.36) | 2.35(2.11, 3.34) | 3.09(2.82, 4.35) |
| **Anterior** | | | | | |
| Esophagus | △RV_25_(%) | -2.88(-4.33, -2.13) | -5.89(-9.74, -4.49) | -8.77(-13.79, -6.81) | -12.80(-17.36, -9.55) |
|  | △D_m_(Gy) | -0.69(-0.81, -0.57) | -1.39(-1.67, -1.11) | -1.92(-2.50, -1.58) | -2.52(-3.42, -2.12) |
| Thyroid | △RV_30_(%) | 0.78(0.46, 1.28) | 1.52(0.79, 2.44) | 2.02(1.20, 3.138) | 2.51(1.55, 3.95) |
|  | △RV_45_(%) | 1.87(0.47, 3.36) | 3.47(1.25, 5.63) | 4.20(2.34, 8.29) | 4.90(2.94, 9.48) |
|  | △D_m_(Gy) | 0.08(0.01, 0.27) | 0.16(-0.01, 0.46) | 0.24(-0.04, 0.60) | 0.29(-0.10, 0.68) |
| Heart | △D_m_(Gy) | -0.30(-0.39, -0.21) | -0.60(-0.71, -0.42) | -0.93(-1.04, -0.62) | -1.22(-1.37, -0.87) |
| Ipsilateral lung | △RV_20_(%) | -1.83(-2.34, -1.45) | -3.11(-4.07, -2.84) | -4.91(-5.96, -4.27) | -6.14(-7.36, -5.60) |
|  | △D_m_(Gy) | -0.63(-0.69, -0.56) | -1.21(-1.39, -1.09) | -1.78(-1.95, -1.63) | -2.36(-2.54, -2.12) |
| LAD | △RV_30_(%) | -3.43(-5.07, -0.03) | -6.07(-8.57, -0.03) | -7.35(-12.34, -0.03) | -7.09(-16.03, -0.03) |
|  | △D_m_(Gy) | -1.47(-1.89, -0.80) | -2.86(-3.31, -1.58) | -4.13(-5.06, -2.49) | -5.22(-6.74, -3.17) |
| **Posterior** | | | | | |
| Esophagus | △RV_25_(%) | 1.69(0.18, 3.54) | 3.76(1.82, 6.12) | 5.62(3.04, 8.99) | 7.35(4.46, 11.82) |
|  | △D_m_(Gy) | 0.63(0.51, 0.74) | 1.20(0.98, 1.59) | 1.74(1.46, 2.09) | 2.24(1.79, 2.68) |
| Thyroid | △RV_30_(%) | -1.29(-2.27, -0.46) | -3.45(-5.28, -0.75) | -4.89(-8.84, -1.33) | -7.05(-12.54, -2.38) |
|  | △RV_45_(%) | -2.73 (-4.70, -1.55) | -5.07(-8.73, -3.16) | -6.88-(11.37, -5.15) | -9.23(-14.09, -6.34) |
|  | △D_m_(Gy) | -0.14(-0.26, -0.05) | -0.39(-0.84, -0.22) | -0.72(-1.28, -0.32) | -1.04(-1.84, -0.39) |
| Heart | △D_m_(Gy) | 0.26(0.20, 0.33) | 0.58(0.42, 0.72) | 0.92(0.67, 1.17) | 1.29(1.10, 1.64) |
| Ipsilateral lung | △RV_20_(%) | 1.48(1.21, 1.66) | 3.04(2.51, 3.34) | 4.62(4.01, 5.10) | 6.35(5.47, 6.93) |
|  | △D_m_(Gy) | 0.61(0.58, 0.67) | 1.23(1.13, 1.44) | 1.74(1.69, 2.07) | 2.34(2.27, 2.76) |
| LAD | △RV_30_(%) | 3.16(0.69, 5.13) | 7.01(2.90, 10.85) | 10.85(5.83, 15.52) | 14.45(10.20, 20.04) |
|  | △D_m_(Gy) | 1.57(0.92, 1.81) | 2.81(2.10, 3.41) | 3.98(3.37, 4.87) | 5.61(4.42, 6.20) |
| **Superior** | | | | | |
| Esophagus | △RV_25_(%) | -0.61(-1.73, 0.04) | -1.17(-2.43, 0.18) | -1.82(-3.55, 0.13) | -2.51(-4.28, -0.32) |
|  | △D_m_(Gy) | -0.35(-0.47, -0.12) | -0.71(-0.91, -0.32) | -1.13(-1.56, -0.51) | -1.57(-2.00, -0.73) |
| Thyroid | △RV_30_(%) | 0.28(0.03, 0.51) | 0.63(0.07, 0.825) | 0.95(0.19, 1.50) | 1.39(0.34, 1.82) |
|  | △RV_45_(%) | 0.41(-0.13, 0.63) | 0.80(-0.39, 1.35) | 1.28(-0.53, 2.11) | 1.76(-0.68, 2.40) |
|  | △D_m_(Gy) | 0.14(0.06, 0.23) | 0.23(0.05, 0.38) | 0.36(0.10, 0.56) | 0.48(0.13, 0.75) |
| Heart | △D_m_(Gy) | -0.08(-0.11, -0.05) | -0.16(-0.21, -0.09) | -0.22(-0.33, -0.13) | -0.30(-0.42, -0.19) |
| Ipsilateral lung | △RV_20_(%) | -1.32(-1.91, -1.07) | -2.52(-3.38, -2.16) | -3.96(-4.56, -3.13) | -5.56(-5.83, -4.24) |
|  | △D_m_(Gy) | -0.46(-0.50, -0.38) | -0.86(-0.92, -0.74) | -1.29(-1.39, -1.14) | -1.72(-1.87, -1.61) |
| LAD | △RV_30_(%) | -0.60(-1.93, -0.03) | -1.35(-3.57, -0.03) | -2.35(-5.36, -0.03) | -3.11(-5.54, -0.03) |
|  | △D_m_(Gy) | -0.26(-0.36, -0.09) | -0.53(-0.76, -0.19) | -0.68(-1.10, -0.27) | -1.17(-1.88, -0.38) |
| **Inferior** | | | | | |
| Esophagus | △RV_25_(%) | 0.80(-0.02, 1.60) | 1.39(-0.43, 2.23) | 1.66(0.06, 2.58) | 1.56(-0.08, 3.14) |
|  | △D_m_(Gy) | 0.31(0.09, 0.43) | 0.62(0.19, 0.83) | 0.87(0.34, 1.20) | 1.14(0.51, 1.53) |
| Thyroid | △RV_30_(%) | -0.11(-0.42, -0.02) | -0.20(-0.46, -0.01) | -0.30(-0.61, 0.08) | -0.35(-0.97, 0.06) |
|  | △RV_45_(%) | -0.59(-1.75, -0.11) | -1.10(-2.46, -0.45) | -1.92(-3.18, -0.39) | -2.69(-4.56, -1.02) |
|  | △D_m_(Gy) | -0.19(-0.25, -0.11) | -0.35(-0.47, -0.21) | -0.51(-0.68, -0.30) | -0.66(-0.87, -0.43) |
| Heart | △D_m_(Gy) | 0.06(0.02, 0.08) | 0.14(0.06, 0.17) | 0.20(0.10, 0.26) | 0.27(0.13, 0.37) |
| Ipsilateral lung | △RV_20_(%) | 1.08(0.79, 1.32) | 2.25(1.70, 2.63) | 3.53(2.86, 4.03) | 4.87(3.84, 5.34) |
|  | △D_m_(Gy) | 0.41(0.36, 0.50) | 0.89(0.77, 0.98) | 1.35(1.17, 1.56) | 1.80(1.60, 2.13) |
| LAD | △RV_30_(%) | 0.21(0.03, 1.22) | 0.94(0.16, 1.95) | 1.53(0.25, 3.04) | 2.04(0.61, 4.18) |
|  | △D_m_(Gy) | 0.24(0.08, 0.50) | 0.53(0.21, 0.97) | 0.87(0.33, 1.39) | 1.13(0.49, 1.85) |

Data are presented as median (25th percentile, 75th percentile). Since the data were not normally distributed, the median and interquartile range (IQR) are used to describe the central tendency and statistical dispersion.

**Supplementary Table2** Dose deviations (perturbed plan vs. original plan) for the esophagus, LAD, heart, and lung are presented under the combination of setup errors in the R, P, and I directions, and for the thyroid under the combination of the R, A, and S directions.

| **OARs** | **Parameter** | **3mm** | **5mm** | **7mm** | **9mm** |
| --- | --- | --- | --- | --- | --- |
| Esophagus | △RV_25_(%) | 13.18(11.45, 15.50) | 22.78(16.93, 27.03) | 27.21(21.37, 37.01) | 32.58(27.18, 45.48) |
|  | △D_m_(Gy) | 3.02(2.72, 3.22) | 5.23(4.80, 5.71) | 7.22(6.18, 8.22) | 8.52(7.28, 9.75) |
| Thyroid | △RV_30_(%) | 6.67(5.92, 7.22) | 9.83(9.08, 10.48) | 13.42(11.89, 14.43) | 17.98(16.61, 20.00) |
|  | △RV_45_(%) | 9.81(7.67, 10.79) | 16.40(14.42, 18.28) | 23.08(21.39, 25.28) | 30.13(26.56, 32.51) |
|  | △D_m_(Gy) | 2.06(1.80, 2.25) | 3.44(3.12, 3.67) | 4.63(4.28, 5.11) | 5.91(5.41, 6.39) |
| Heart | △D_m_(Gy) | 1.04(0.98, 1.11) | 1.60(1.44, 1.74) | 2.14(1.89, 2.39) | 2.61(2.42, 2.96) |
| Ipsilateral lung | △RV_20_(%) | 6.49(6.22, 6.67) | 10.06(9.53, 10.36) | 14.07(13.46, 14.55) | 20.00(19.12, 20.58) |
|  | △D_m_(Gy) | 1.39(1.36, 1.45) | 2.25(2.15, 2.46) | 2.96(2.91, 3.29) | 3.66(3.59, 4.08) |
| LAD | △RV_30_(%) | 8.17(5.70, 10.14) | 14.03(9.92, 17.87) | 20.30(15.28, 24.97) | 28.10(23.85, 33.69) |
|  | △D_m_(Gy) | 2.35(1.70, 2.59) | 3.83(3.12, 4.43) | 5.20(4.59, 6.09) | 6.93(5.74, 7.52) |

Data are presented as median (25th percentile, 75th percentile). Since the data were not normally distributed, the median and interquartile range (IQR) are used to describe the central tendency and statistical dispersion. R: Right. P: Posterior. I: Inferior
